# Clinical Pharmacology of Hormonal Emergency Contraceptive Pills

**DOI:** 10.1155/2018/2785839

**Published:** 2018-10-04

**Authors:** Celia M. J. Matyanga, Blessing Dzingirai

**Affiliations:** School of Pharmacy, College of Health Sciences, University of Zimbabwe, Harare (263), Zimbabwe

## Abstract

Emergency contraceptives play a major role in preventing unwanted pregnancy. The use of emergency contraceptives is characterized by myths and lack of knowledge by both health professionals and users. The main objective of this paper is to summarize the clinical pharmacology of hormonal methods of emergency contraception. A literature review was done to describe in detail the mechanism of action, efficacy, pharmacokinetics, safety profile, and drug interactions of hormonal emergency contraceptive pills. This information is useful to healthcare professionals and users to fully understand how hormonal emergency contraceptive methods work.

## 1. Introduction

Unintended pregnancies are widespread and may be due to different reasons. In the year 2012, there were 213 million pregnancies globally and, of these, 85 million (almost 40%) were unintended. Of the unintended pregnancies, 50% ended in abortion, 38% resulted in unplanned births and 13% led to miscarriages [1]. Abortion in many African countries is prohibited and unsafe, contributing 9% of all maternal deaths [2]. This is where emergency contraceptives play a major role in preventing unwanted pregnancies. Emergency contraceptives (EC) are methods that can be used to prevent unwanted pregnancy if they are used within a specified time. They are also known as postcoital contraception or “morning–after” pills if they are oral tablets. Many myths surround the use of emergency contraception. These myths include the following: they can only be used the morning after, they are the same as abortion, repeated use can result in infertility, there are no side effects associated with using them, they protect against sexually transmitted diseases, among others [3]. The aim of this paper is to demystify these myths and guide pharmacists, prescribers, other health professionals, and even consumers on the proper use of emergency contraceptives.

### 1.1. Indications for Emergency Contraception

Several circumstances may warrant the use of emergency contraception. ECs can be used in the following situations: after having unprotected sexual intercourse, when a woman has incorrectly used regular contraceptives, in the event of sexual assault, or even in situations where a condom has burst, slipped, or been used incorrectly [4, 5]. The available methods of emergency contraception include using emergency contraception pills (ECPs), the Yuzpe method, or the copper-bearing intrauterine devices (IUDs). The Yuzpe method was named after the Canadian physician who first described the regimen [6]. This paper will focus its discussion on the clinical pharmacology of the hormonal emergency contraceptive pills, that is, the ECPs and Yuzpe method.

## 2. Methods

A thorough literature review was conducted to describe in detail the mechanism of action, efficacy, pharmacokinetics, safety profile, and drug interactions of hormonal emergency contraceptive pills. Literature review was conducted through searching PubMed, Medline, and Google Scholar. Google webpage was used as the search engine for information outside published articles. The following words were used for literature search in Google webpage: “emergency contraception”, “emergency contraception clinical pharmacology”, “levonorgestrel package insert”, and “ulipristal package insert”. From the search results, relevant websites were viewed to compile the paper. From the health databases, the following words were used as search hits: “emergency contraception”, “levonorgestrel emergency contraception”, “emergency contraception pills”, “ulipristal contraception”, “safety emergency contraception”, and “post coital contraception.” The most relevant and where applicable current articles were selected for discussion. The literature search was conducted between January 2018 and July 2018.

## 3. Results and Discussion

### 3.1. Types of EC

Emergency contraception pills (ECP) include levonorgestrel (LNG) and ulipristal acetate. Levonorgestrel is a synthetic progestin taken orally as a single dose of 1.5 mg strength. Alternatively, LNG can be taken in two doses of 0.75 mg each separated by 12 hours [4, 7]. Studies have shown that a single dose of 1.5 mg is as effective as two 0.75 mg doses taken 12 hours apart [8, 9]. The LNG method is widely used and is the gold standard emergency contraception regimen [10, 11]. Another option is to use ulipristal acetate, which is a selective progesterone receptor modulator. Ulipristal is taken as a single dose of 30 mg [11]. The United States Food and Drug Administration (US FDA) approved ulipristal for emergency contraception in August 2010 [12]. However, it is only available in a few developed countries.

The Yuzpe method uses estrogen combined with levonorgestrel. The pills are taken in two divided doses. Each dose must contain estrogen (usually 100–120 mcg ethinylestradiol) and progestin (either 0.50–0.60 mg levonorgestrel or 1.0–1.2 mg norgestrel) [4, 6]. For the Yuzpe method, the ordinary birth control pills are used in specified combinations as emergency contraception. Depending on the brand available and the concentrations of the estrogen/ progestin, each dose consists of 4, 5, or 6 pills. The regimen is one dose followed by a second dose 12 hours later [6].

### 3.2. Mechanism of Action

In emergency contraception, studies have shown that levonorgestrel works by preventing or delaying ovulation and impairing luteal function [13–15]. LNG may also increase the thickness of the cervical mucus or affect sperm migration and function in the genital tract, thereby preventing fertilization of an egg [16]. Some studies have found no effect of levonorgestrel on the endometrium, the quality of cervical mucus, nor the penetration of spermatozoa in the uterine cavity [17–19]. The exact mechanism of action remains unclear, and this knowledge gap could be addressed by further research in this area.

Ulipristal acetate is thought to inhibit or delay ovulation. A clinical trial showed that it could delay ovulation for 24–48 hours even on the day of the luteinizing hormone (LH) peak. When ulipristal was given before or immediately after the LH surge, it inhibited 100% of the follicular ruptures [20]. Other mechanisms include reducing the endometrial thickness, delayed endometrial maturation, and alterations in the progesterone–dependent markers required for implantation. These effects may subsequently inhibit implantation because the uterus will be less receptive to the trophoblast [21].

Combined ECP work by inhibiting implantation of a fertilized egg [22]. Other postulated mechanisms include delaying or suppressing ovulation, interfering with corpus luteum function and making changes in the endometrium that prevents implantation [23–25].

### 3.3. Efficacy of ECP

Hormonal emergency contraception has been shown to be effective when used up to 72 hours after unprotected intercourse. However, the earlier the treatment begins, the more effective it is [6, 26]. In contrast, this does not apply to ulipristal. Within 72 hours of unprotected sex, ulipristal is at least as effective as levonorgestrel and estimated to be 98.2% to 99.1% effective [27, 28]. Ulipristal maintains consistent effectiveness up to 5 days (120 hours) after unprotected intercourse, whilst the effectiveness of levonorgestrel declines when given more than 48 hours after unprotected sexual intercourse [8, 28, 29]. Within 72 to 120 hours, ulipristal is more effective than levonorgestrel [27].

The Yuzpe regimen of emergency contraception is reported to be 97% to 98% effective in preventing pregnancy [26]. WHO conducted a double-blinded randomized trial of 1 998 women from 14 countries comparing the use of levonorgestrel with the Yuzpe combination. The levonorgestrel regimen was found to be more effective at preventing pregnancy. The LNG regimen decreased the average expected pregnancy rate by 85% (95% confidence interval [CI] 74:93) compared to the Yuzpe regimen rate of 57% (95% CI 39:71) [30].

### 3.4. Pharmacokinetics

Levonorgestrel does not undergo “first–pass” metabolism and has 100% bioavailability [7]. This makes it effective when administered orally as a tablet. After a single dose administration of levonorgestrel, maximum plasma concentrations of 19.1ng/mL were reached at a median of 1.7 hours (range 1–4 hrs.) No studies have evaluated the effect of food on the rate and the extent of levonorgestrel absorption following single oral administration. Studies have shown that the apparent volume of distribution (Vd) of levonorgestrel is approximately 1.8 L/kg. The elimination half-life of levonorgestrel after single dose administration (0.75 mg) was 27.5 + 5.6 hours. These pharmacokinetic parameters facilitate the single and 12-hour dosing of LNG.

Levonorgestrel is highly protein bound (97.5 to 99%), mainly to the sex hormone binding globulin (SHBG) and, to a lesser extent, serum albumin. Any displacement of LNG to the bound protein could potentially cause side effects, hence the need to monitor concurrent medications. Approximately 45% of levonorgestrel and its metabolites are excreted in the urine and about 32% are excreted in feces, mostly as glucuronide conjugates [7].

Ulipristal is rapidly absorbed after a single 30 mg dose is administered orally. Peak plasma concentration of 176 ± 89 ng/ ml occurs approximately 0.5–3hours after oral ingestion [11]. Ulipristal can be taken with or without food. When administered together with a high fat meal, studies showed that there was a 40–45% lower mean Cmax (maximum or peak serum concentration), a delayed time to maximum concentration (tmax), and 20–25% higher mean area under the drug concentration curve from time zero to infinity (AUC_0-*∞*_) in comparison to the fasting state. Ulipristal is highly bound to plasma proteins (>98%) including albumin, high-density lipoproteins, and alpha-1-acid glycoprotein. It is extensively metabolized in the liver via the CYP3A4 pathway. The main metabolites are monodemethylated and didemethylated metabolites. The monodemethylated metabolite is pharmacologically active. The terminal half-life of ulipristal in plasma is 32.4 ± 6.3 hours [31].

### 3.5. Safety Profile

The side effects of hormonal emergency contraceptives are uncommon and mild and generally similar to those experienced by women using regular oral contraceptive pills. The levonorgestrel regimen is significantly better tolerated than the Yuzpe combined regimen. The double-blinded randomized trial by WHO showed that only 23.1% of women who used progestin alone experienced nausea compared with 50% of women in the Yuzpe regimen. In addition, only 5.6% of women in the progestin group reported vomiting compared with 18.8%. In both groups menstrual patterns were similar [30]. Therefore, the progestin-only pills have a favorable safety profile than the combined ECP.

Ulipristal is also well tolerated and the adverse effects are mild and generally self-limiting. The side effect profile for ulipristal is similar to that of levonorgestrel emergency contraception. Data from clinical trials reported the following symptoms as more frequent: nausea, headache, abdominal pain, dizziness, back pain, and dysmenorrhea [27].

Other uncommon side effects associated with both the progestin and the Yuzpe regimens include headache, bloating, and uterine cramps [32]. Taking each dose of emergency contraceptive with food and using antiemetics 30 minutes before may reduce the gastrointestinal side effects.

### 3.6. Effects on Pregnancy

Some authors have supported the view that emergency contraception is a form of abortion arguing that preovulatory administration of levonorgestrel as an emergency contraceptive has significant potential to work via abortion [14, 33]. Conventionally, any drug or device that acts after implantation is termed an abortifacient rather than a contraceptive [34]. Since the mechanism of action for emergency contraceptives does not involve dislodging an implanted fetus, they would conventionally not be termed abortifacients.

When ulipristal is administered to a pregnant woman, the risks to the fetus are unknown. When it was repeatedly administered to pregnant rats, embryo fetal loss was noted in all pregnant rats after 12 days of dosing. Repeated administration for 13 days in pregnant rabbits resulted in embryo fetal loss in 50% of the rabbits. When it was administered daily to pregnant monkeys for 4 days within the first trimester, ulipristal caused pregnancy termination in two of the five animals. There were no malformations of the surviving fetuses in these studies [12]. Since there are no studies or data in humans, we have to extrapolate data from animal studies. Hence, ulipristal carries a warning that it is contraindicated during an existing or suspected pregnancy. There is limited information regarding use of regimens containing levonorgestrel and/oestrogen on future pregnancy. A study in China compared pregnancy outcomes between women who had used LNG against a group that had not used LNG during the conception cycle. The results showed that there was no association between using LNG as an emergency contraception and the risk of congenital malformations or pregnancy complications [35]. There is a knowledge gap on the effects of using emergency contraception and future pregnancies.

### 3.7. Potential Interactions

Drugs, foods, or herbs that induce the cytochrome P450 (CYP450) enzymes, including CYP3A4, that metabolize progestins, and estrogens, may decrease their plasma concentrations. This may decrease the effectiveness of progestins and estrogens. Examples may include St. John's Wort, carbamazepine, rifampicin, phenytoin, and griseofulvin [36]. Coadministration of levonorgestrel with HIV protease inhibitors or with nonnucleoside reverse transcriptase inhibitors has shown significant changes (increase or decrease) in the plasma levels of levonorgestrel. CYP3A4 inhibitors such as itraconazole or ketoconazole may increase plasma concentrations of ulipristal [7, 12]. Studies conducted* in vitro* showed that ulipristal does not induce or inhibit the activity of CYP450 enzymes; however, it may be an inhibitor of P-glycoprotein (P-gp) at clinically relevant concentrations. Therefore, it is not advisable to administer ulipristal with P-gp substrates (e.g., digoxin, colchicine, and fexofenadine) as it may increase the concentration of P-gp substrates [12].

Since ulipristal is an antiprogestin; there are implications on starting progestin-containing hormonal contraceptives immediately after taking it. Two randomized studies found that using ulipristal and progestin containing hormonal contraceptives resulted in no significant differences in time to ovarian quiescence or cervical mucus penetrability. Both studies showed that using a progestin, containing oral contraceptive immediately after ulipristal, reduces the efficacy of ulipristal. This impairs the ability of ulipristal to delay ovulation [37, 38]. This is because ulipristal and the progestin component of hormonal contraceptives both bind to the progesterone receptor; thus coadministration may impair the ability of ulipristal to delay ovulation. If a woman wants to use hormonal contraception after using ulipristal, she should do so 5 days after the intake of ulipristal and she should use a reliable barrier method until the next menstrual period [12, 32].

## 4. Conclusion

Emergency contraceptives, commonly known as the “morning–after” pill, are effective in preventing unplanned pregnancy. The term “morning–after” is misleading; emergency contraceptives should be initiated sooner than the morning after, that is, immediately after unprotected intercourse. For both the levonorgestrel containing and combined emergency contraceptive pills, efficacy decreases after 72 hours; thus the sooner they are taken the better the chances of preventing unplanned pregnancy. Ulipristal, on the other hand, has no decreased efficacy and works for up to 120 hours. The progestin containing regimens are more effective and better tolerated than the estrogen—containing regimens. Emergency contraceptives should not be used as a regular form of contraception and do not protect against sexually transmitted infections, including HIV/AIDS.
